# Surgical treatment for pelvic giant cell tumor: a multi-center study

**DOI:** 10.1186/s12957-016-0862-0

**Published:** 2016-04-05

**Authors:** Kai Zheng, Xiuchun Yu, Yongcheng Hu, Zhen Wang, Sujia Wu, Zhaoming Ye

**Affiliations:** Second Military Medical University, Shanghai, China; Department of Orthopaedics, The General Hospital of Jinan Military Commanding Region, Jinan, Shandong China; Department of Bone Oncology, Tianjin Hospital, Tianjin, China; Department of Orthopaedics, Xijing Hospital, Fourth Military Medical University, Xian, Shannxi China; Department of Orthopaedics, Jinling Hospital, Clinical School of Medical College, Nanjing University, Nanjing, Jiangsu China; Department of Orthopaedics, Second Affiliated Hospital, School of Medicine, Zhejiang University, Hangzhou, Zhejiang China; Giant Cell Tumor Group of China (GTOC), Beijing, China

**Keywords:** Giant cell tumor, Pelvis, Surgical treatment

## Abstract

**Background:**

The purpose of this study was to discuss the clinical results which related to the location of giant cell tumors (GCTs) in the pelvis so as to determine the ideal surgical treatment protocol.

**Methods:**

We report 29 cases who accepted surgical treatment from five clinical centers during the last 12 years. All patients were divided into three groups according to tumor locations, and they were also classified into two groups in light of surgical treatments. The parameters for outcome evaluation consisting of general condition, surgical complications, local disease control, and Musculoskeletal Tumor Society (MSTS) 93 functional score had been analyzed, respectively.

**Results:**

Surgical treatment in the acetabular area usually resulted in postoperative complications and poor function. One patient who accepted intralesional surgery and two who accepted wide resection had local recurrence. The mean functional score was 25.4 for the 8 patients who received intralesional surgery and 21.9 for the 21 patients who received wide resection. Surgical complications occurred in 1 patient who underwent intralesional surgery and the other 6 patients who underwent wide resection.

**Conclusions:**

We conclude that surgical treatment of pelvic GCTs in the acetabular area is difficult to select as it is always accompanied by complications and poor function. Compared to wide resection, intralesional surgery combined with a meticulous preoperative planning may lower the recurrence rate and obtain favorable postoperative functional results.

## Background

Giant cell tumors (GCTs) of the bone represent 3–8 % of all primary bone tumors [1–3], most of which occur at the ends of long bones. Accounting for only 1.5 to 6.1 % of bone GCTs [4–6], pelvic GCTs are rare lesions and no more than 100 cases have been reported in the past five decades before 2004 [7]. In the limited number of literature, Guo et al. reviewed 27 cases with pelvic GCTs, which is the largest series that had ever been reported [8]. To our best knowledge, a multi-center clinical study of GCTs has not been available in any publications. In this study, we collected the data of 29 cases from five clinical institutions to analyze the correlation between the location of GCTs in the pelvis and the clinical results and to discuss the selection of surgical treatment options.

There is still no widely held consensus in respect to the ideal surgical treatment protocol for giant cell tumors of the pelvis owing to their infrequent occurrence. Due to the complex anatomy of the pelvis and mostly delayed presence in clinical institution, the treatment for pelvic GCTs might be challenging without a standard treatment protocol for consultation. Treatment options for pelvic GCTs include denosumab [9], serial embolization [10], interferon [11], radiation therapy [12], and intralesional curettage or wide resection [7, 13–16]. Although some primary treatments are favorable for GCT control, it is still necessary to perform tumor resection if the lesion has become resectable. Strengths of intralesional curettage include preservation of pelvic integrity and avoidance of nerve disturbance, but it is also deemed to increase the risk of local recurrence, especially for aggressive benign tumors [7, 13–16]. Although wide resection is reported to minimize the chance of local recurrence, this procedure seems to have certain disadvantages such as long-time operation, increased risk of nerve injury and infection, and prosthesis-related complications from hip reconstruction [17–19]. Some suggestions have been given in literatures by retrospective analysis of their own treated patients, but it is hard to say the treatments are not influenced by their preference. Given the multi-center study of pelvic GCTs has not been reported in any literatures, in this study, we reviewed 29 cases treated by five bone tumor experts from five institutions, which hopefully provides information of great value to orthopedic surgeons and assist them to select the optimal treatment protocol from various options.

## Methods

Five hundred thirteen patients with histologically benign GCT of the bone were treated at these five institutions from 2001 to 2013, of which 29 patients who presented with pelvic GCT were retrospectively reviewed. In this study, we included patients with the following criteria: (1) pathological diagnosis of GCT was definite; (2) GCT involved the pelvis but not the sacrum; (3) no prior treatments of the tumor; and (4) complete clinical, radiographic, and pathologic records. Most patients were aged between the third and fourth decades of life at first diagnosis, with a mean age of 39.2 years (range 15–57 years). In this series, there were 14 males and 15 females with a mean follow-up period of 59 months (range 18–156 months) (Table 1). Eight patients were treated with intralesional surgery while 21 underwent wide resection. The patients’ data was collected from patient records, surgical protocols, and histological and radiological findings. The last follow-up was done via follow-up exams after surgery or telephone contact.

**Table 1:** Clinical data and surgical results for 29 patients with GCTs involving the pelvis

No.	Gender/age (years)	Location^a^	Grade^b^	Treatment	Reconstruction	Complication	Follow-up (months)	Function (MSTS 93)	Recurrence or metastasis
1	F/39	I + II	III	Wide resection	Rod fixation + THA		24	19	Recurrence
2	M/34	I + II	III	Wide resection	Rod fixation + THA	Wound healing disturbance	136	22	No
3	F/33	I	II	Wide resection	No		63	26	No
4	F/42	I + II	III	Wide resection	Rod fixation + THA	Wound healing disturbance	12	17	Recurrence
5	M/47	III	I	Wide resection	No		36	27	No
6	M/42	II + III	III	Wide resection	Rod fixation + THA		22	16	No
7	F/43	II	II	Intralesional curettage	No	Limb shortening	156	18	No
8	F/25	I	II	Wide resection	No		140	25	No
9	M/37	I + II	III	Intralesional curettage	Cement		6	23	Recurrence
10	M/54	III	II	Wide resection	No		56	22	No
11	M/57	III	I	Wide resection	No		53	23	No
12	M/41	II + III	III	Wide resection	Cement	Delayed infection	49	18	No
13	F/41	II	III	Wide resection	No	Dislocation	38	14	No
14	F/15	II + III	III	Microwave + curettage	Bone graft		148	28	No
15	F/34	II	III	Microwave + curettage	Bone graft		37	26	No
16	F/42	III	III	Microwave + curettage	Bone graft		21	28	No
17	M/50	III	II	Wide resection	No		18	26	No
18	M/27	III	III	Wide resection	No		30	24	No
19	M/46	III	II	Wide resection	No		37	26	No
20	M/37	II + III	III	Wide resection	Rod fixation + THA	Wound healing disturbance	18	21	No
21	F/50	I	II	Wide resection	Pelvic ring reconstruction		22	24	No
22	F/41	I	II	Wide resection	No		26	22	No
23	M/33	I	III	Wide resection	Pelvic ring reconstruction		28	20	No
24	F/30	I	II	Intralesional curettage	Cement		93	24	No
25	F/39	I	II	Intralesional curettage	No		85	28	No
26	M/32	I	III	Wide resection	Pelvic ring reconstruction	Wound healing disturbance	77	21	No
27	M/34	I	III	Wide resection	No		60	22	No
28	F/38	III	II	Intralesional curettage	No		56	28	No
29	F/55	I	II	Wide resection	No		28	24	No

*M* male, *F* female, *THA* total hip arthroplasty

^a^According to the classification system of Enneking and Dunham

^b^According to the Campanacci grading system

The research was carried out according to the principles set out in the Declaration of Helsinki 1964 and all subsequent revisions. Informed consent was obtained, and the relevant institutional review board (Jinan Military General Hospital Ethics Committee) had approved the study.

The diagnosis of GCT was established based on the clinical data and imaging studies and confirmed by needle biopsy or open biopsy before surgery as well as pathology examination after surgery. In accordance with the classification system for pelvic tumors by Enneking and Dunham [17], further modified by Sanjay et al. [16], the locations of tumor were divided into three types: type I (ilium), type II (acetabulum), and type III (pubis/ischium). This study included type I lesions in 10 patients, type II in 11 patients, and type III in 8 patients. Radiographical classification system of Campanacci [20] categorizes the lesion into three grades in which grade I indicates an intraosseous lesion, grade II denotes an intraosseous lesion with cortical thinning and expansile borders, and grade III refers to a lesion extending extraosseously and forming a soft tissue mass. There were 5 patients with grade I lesions, 15 patients with grade II lesions, and 9 patients with grade III lesions included in this series. The clinical results were statistically analyzed in light of different GCT locations in the pelvis.

**Fig. 1:**
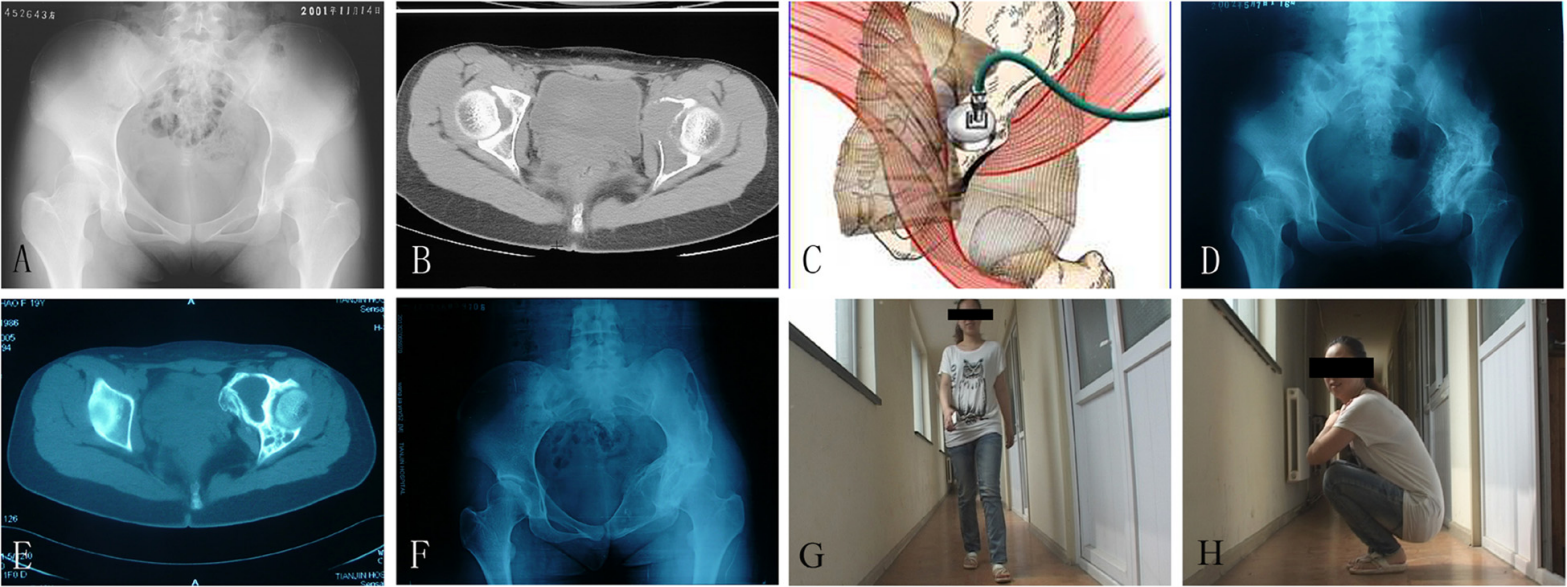
**a** Plain film of a 15-year-old female demonstrates an osteolytic lesion in the left periacetabular region. **b** CT scan presents the tumor involvement in the acetabulum, but the acetabulum cartilage and bone below it can be preserved. **c** Sketch illustrates that microwave inactivation was employed for this patient before intralesional curettage. **d** Plain film at 6 months after operation shows a suspicious lesion in the left pubis. No treatment was performed as there is no presentation of pain or other discomfort. **e** CT scan at 4 years after operation manifests that the intumescent lesion in the left pubis has mineralized edge. **f** Plain film at 11 years after operation shows no recurrence. **g**, **h** The patient had good function without any discomfort in the last follow-up

**Fig. 2:**
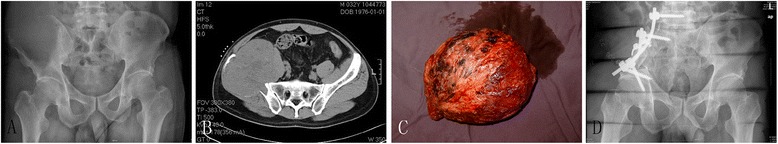
Case presentation of a 32-year-old male presented a persistent pain of the left ilium for 3 months. **a** Osteolytic lesions can be observed in the left ilium on the plain film. **b** CT scan shows the tumor involvement in the ilium where a huge soft tissue mass formed. **c** The tumor was resected widely, and the inferior cut line was at the normal bone above the acetabulum. **d** Screws and rods were used for reconstruction of the pelvic ring after tumor resection

**Fig. 3:**
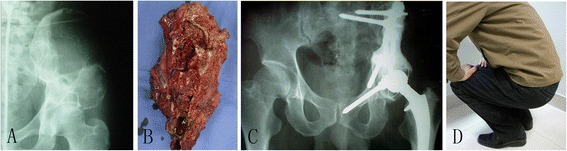
Case presentation of a 34-year-old man who mainly complained about pain in the left hip area for 5 months which was aggravated over the previous 1 week. **a** Osteolytic lesions can be observed in the left ilium and periacetabular region on the plain film. **b** The tumor was resected widely, and the cut line was at the normal bone. **c** A plain film at 2 years after operation shows no recurrence. **d** The patient has good function without any discomfort in the last follow-up

The treatment regimens were classified into group A and group B, and group A had been further divided into two subgroups. Group A1 included 5 patients treated by intralesional curettage, of which two cases applied bone cement to fill defect after curettage. Group A2 included 3 patients treated by microwave inactivation for the GCT lesion before intralesional curettage, after which defects were filled with autograft and allogeneic bone graft (Fig. 1). Twenty-one patients treated by wide resection were included by group B, of which 3 patients involving region I underwent reconstruction of the pelvic ring after resection (Fig. 2) and 5 patients involving region II underwent rod fixation and total hip arthroplasty (Fig. 3). One patient in group B involving region II accepted bone cement filling because of small bone defect, while another patient involving region II accepted femoral head exclusion after resection of pelvic tumors around the acetabulum. The other 11 patients in group B including 5 patients in region I and 6 patients in region III did not receive any reconstruction after resection. The embolization procedure was not performed in some cases as the tumor side of the common iliac artery was temporarily blocked by a vascular clamp to control bleeding during surgery. The effectiveness has been documented that the common iliac artery was mobilized and encircled with nylon tape for temporary occlusion during removal of the tumor [21]. Patients who accepted microwave inactivation before curettage did not have blood transfusion while most of the others had. Adjuvant therapy such as chemotherapy or radiotherapy was not used in the initial treatment in this series. One patient was treated with radiotherapy for the recurrent tumor after surgery.

All patients had been requested to reexamine every month for half a year after surgery, every 3 months in 0.5–2 years after surgery, and annually after 2 years. The complications and local disease control were recorded each time. Local recurrence was suspected initially by evidence of new bone involvement assessed by radiographs or computerized tomography (CT), and a biopsy was further performed to confirm the suspicion. Musculoskeletal Tumor Society (MSTS) 93 score [22] had been used for functional evaluation at the last follow-up in our study. The MSTS 93 score measures patient activity, including pain, function, emotional acceptance, supports, walking ability, and gait. Each of these six variables was assessed on a five-point scale, giving a maximum score of 30 points. To some extent, higher MSTS score signifies better functional results.

## Results

### Group A1: intralesional curettage (5 cases)

Group A1 was the simplest treatment regimen that intralesional curettage was followed by bone cement filling of the defect (cases 9 and 24) or not (cases 7, 25, and 28). Three patients (cases 24, 25, and 28) demonstrated good functions and were disease-free at follow-ups. One patient (case 9) with ilium and acetabulum lesion who was primarily treated by intralesional curettage and cementation suffered local recurrence 6 months after surgery. The recurrence was confirmed by biopsy, and he refused additional treatments although there is a possibility of reoperation. He died of unclear cause without any imaging and pathology 2 months later. One patient (case 7) with acetabulum lesion underwent limb shortening. At 3-year follow-up, the patient was free from pain, but she limped and leaned on a cane for longer walks due to the 5-cm shortening. As the shortening gradually worsened, total hip arthroplasty was undertaken for her at 5 years after primary surgery.

### Group A2: microwave inactivation for the GCT lesion before intralesional curettage (3 cases)

Three patients (cases 14, 15, and 16) with partial acetabulum lesion underwent microwave inactivation before intralesional curettage, after which defects were filled with autograft and allogeneic bone graft. They remained disease-free and had no functional limitation at follow-ups. No recurrence and complications were found in this group.

### Group B: wide resection (21 cases)

The 5 patients with tumors of region I (cases 3, 8, 22, 27, and 29) were treated by wide resection of the iliac tumor without reconstruction whereas the other 3 patients with tumors of region I (cases 21, 23, and 26) were treated by wide resection with pelvic ring reconstruction. The 6 patients with tumors of region III (cases 5, 10, 11, 17, 18, and 29) underwent wide resection without reconstruction. The 5 patients with tumors of region II (cases 1, 2, 4, 6, and 20) received wide resection with rod fixation and total hip arthroplasty whereas the other 2 patients (cases 12 and 13) received wide resection without reconstruction. One patient (case 4) who had wide resection suffered local recurrence at 12 months after surgery, and she accepted hemipelvectomy after biopsy confirmation as well as radiation therapy for pulmonary metastasis 2 months after hemipelvectomy. Unfortunately, she died of metastasis. One patient (case 1) who underwent resection of the tumor in regions I and II recurred in region III at 18 months after surgery, and she died of unclear cause at half a year after recurrence without additional treatments and imaging examination. Four patients (cases 2, 4, 20, and 26) with wound healing problems were managed by debridement with eventual healing between 4 and 6 weeks. Dislocation of the hip occurred in 1 patient (case 13) with femoral head exclusion after resection of pelvic tumors around the acetabulum, and she got local stability after long periods of bed rest. This patient could walk with crutches with no pain at the most recent follow-up. One patient (case 12) with a delayed infection eventually healed after wound debridement for three times and long-term antibiotic use.

GCT locations in the pelvis seem to be an influence factor regarding the surgical complications and MSTS functional score. Surgical complications were much more common, and the MSTS functional score was lower when the GCTs involved the acetabulum than those in the ilium or pubis/ischium (*p* = 0.010, *p* = 0.005) (Table 2).

**Table 2:** The statistic analyses of 29 pelvic GCT patients basing on the location group

Categories	Type I^a^	Type II^a^	Type III^a^	Total	*p*
Number, *n* (%)	10 (34.5)	11 (37.9)	8 (27.6)	29 (100)	–
Age, year, means (SD)	37.2 (9.3)	36.8 (7.9)	45.1 (9.5)	39.2 (9.3)	0.106
Age group, *n* (%)					0.175
<20	0 (0)	1 (9.1)	0 (0)	1 (3.4)	
20~29	1 (10.0)	0 (0)	1 (12.5)	2 (6.9)	
30~39	6 (60.0)	5 (45.5)	1 (12.5)	12 (41.4)	
40~49	1 (10.0)	5 (45.5)	3 (37.5)	9 (31.0)	
≥50	2 (20.0)	0 (0)	3 (37.5)	5 (17.2)	
Sex, *n* (%)					0.160
Male	3 (30.0)	5 (45.5)	6 (75.0)	14 (48.3)	
Female	7 (70.0)	6 (54.5)	2 (25.0)	15 (51.7)	
Treatment, *n* (%)					0.691
S (IL)	2 (20.0)	4 (36.4)	2 (25.0)	8 (34.5)	
S (W)	8 (80.0)	7 (63.6)	6 (75.0)	21 (31.0)	
Follow-up, months, means (SD)	62.2 (38.0)	75.5 (59.9)	38.3 (15.3)	59.0 (42.7)	0.215
Recurrence, *n* (%)					0.065
Exist	0 (0)	3 (27.3)	0 (0)	3 (10.3)	
None	10 (100)	8 (72.7)	8 (100)	26 (89.7)	
Complication, *n* (%)					0.010
Exist	1 (10.0)	6 (54.5)	0 (0)	7 (24.1)	
None	9 (90.0)	5 (45.5)	8 (100)	22 (75.9)	
MSTS, means (SD)	23.6 (2.4)	20.2 (4.3)	25.5 (2.3)	22.8 (3.8)	0.005

*S (IL)* intralesional curettage, *S (W)* wide resection

^a^According to the classification system of Enneking and Dunham

The mean MSTS functional score at the last follow-up was 22.8 (range 14–28) with a mean score of 25.4 for 8 patients who underwent intralesional surgery and 21.9 for 21 patients treated with wide resection. Some parameters were used for statistical analyses between group A (intralesional surgery) and group B (wide resection), but significant difference could only be found in the MSTS functional score (*p* = 0.024) (Table 3).

**Table 3:** The statistic analyses of 29 pelvic GCT patients basing on the treatment group

Categories	S (IL)	S (W)	Total	*p*
Number, *n* (%)	8 (27.6)	21 (72.4)	29 (100)	–
Age, year, means (SD)	34.8 (9.0)	41.0 (9.0)	39.2 (9.3)	0.732
Age group, *n* (%)				0.190
<20	1 (12.5)	0 (0)	1 (3.4)	
20~29	0 (0)	2 (9.5)	2 (6.9)	
30~39	5 (62.5)	7 (33.3)	12 (41.4)	
40~49	2 (25.0)	7 (33.3)	9 (31.0)	
≥50	0 (0)	5 (23.8)	5 (17.2)	
Sex, *n* (%)				0.035
Male	1 (7.1)	13 (92.9)	14 (48.3)	
Female	7 (46.7)	8 (53.3)	15 (51.7)	
Location,^a^ *n* (%)				0.691
I	2 (25.0)	8 (38.1)	10 (34.5)	
II	4 (50.0)	7 (33.3)	11 (52.4)	
III	2 (25.0)	6 (28.6)	8 (27.6)	
Follow-up, months, means (SD)	85.1 (52.1)	49.3 (35.4)	59.0 (42.7)	0.056
Recurrence, *n* (%)				0.814
Exist	1 (12.5)	2 (9.5)	3 (10.3)	
None	7 (87.5)	19 (90.5)	26 (89.7)	
Complication, *n* (%)				0.635
Exist	1 (12.5)	6 (28.6)	7 (24.1)	
None	7 (87.5)	15 (71.4)	22 (75.9)	
MSTS, means (SD)	25.4 (3.6)	21.9 (3.5)	22.8 (3.8)	0.024

*S (IL)* intralesional curettage, *S (W)* wide resection

^a^According to the classification system of Enneking and Dunham

## Discussion

Giant cell tumors of the bone rarely affect the pelvis, with an incidence of only 1.5 to 6.1 % of bone GCTs [4–6]. Only 73 pelvic lesions had reported in literatures from 1949 to 1999 [7], and the largest number of cases with pelvis GCTs presented in one study was no more than 30. In this study, 29 patients with pelvis GCTs from five institutions had been collected for analysis. To our knowledge, it is the largest number of cases in the existing literatures as well as the only multi-center study on pelvis GCTs.

The local recurrence rate for giant cell tumors of the pelvis seems to be higher than that of any other location in the skeleton for western or eastern [23, 24], due to the complex anatomy and the large size these lesions can attain before diagnosis. The local recurrence rate can be as high as 43 % for therapeutic options other than wide resection [7, 13–16, 25]. Sanjay et al. [16] reported that 3 of 15 patients with pelvic GCTs had local recurrence after intralesional surgery, whereas no one recurred in 2 patients who underwent wide resection. Balke et al. [13] reported that 1 of 16 patients with pelvic GCTs suffered local recurrence after intralesional surgery, whereas none of 3 patients with wide resection recurred. Guo et al. [8] reported that 4 of 13 patients with pelvic GCTs had local recurrence after intralesional surgery, whereas 0 of 14 patients suffered recurrence after wide resection. In our study, 2 of the 21 patients who underwent wide resection had local recurrences, whereas one of 8 patients who underwent intralesional surgery had local recurrence. No significant difference in recurrence was found between intralesional surgery and wide resection (*p* = 0.814). It is generally accepted that wide resection can reduce the risk of recurrence because the adjacent tissue can be resected with the tumor to achieve an adequate resection margin. For some pelvic GCTs, wide resection is hard to perform owing to the complex anatomic structures in the pelvis. Intralesional surgery is an adequate option for some patients based on careful preoperative planning. Initial surgical treatment is of great importance to patients on account of the fact that recurrence of the tumor may make it unresectable; as a consequence, it is vital for patients with pelvic GCTs to be operated by surgeons with sufficient knowledge of bone tumor. The tumor size and Campanacci classification should be taken into account in the preoperative plan. If the tumor is not limited to one region defined by the Enneking and Dunham classification system for pelvic tumors, we believe wide resection could be a reasonable choice. If the Campanacci classification is grade III that the bone walls are destroyed by the tumor and a large soft tissue mass forms, wide resection is recommended to ensure a safe margin.

Treatment options for pelvic GCTs include denosumab, serial embolization, interferon, radiation therapy, and surgical treatment. Denosumab is a fully humanized monoclonal antibody against the RANK ligand which has demonstrated significant activity in patients with unresectable or recurrent GCTs in the bone [9]. Denosumab was approved by the FDA for the treatment of adults and skeletally mature adolescents with GCT of the bone that is unresectable or where surgical resection is likely to result in severe morbidity in 2013. However, patients did not accept the denosumab treatment in China because the denosumab was not approved by CFDA and the patients’ agreement was hard to get. Although serial embolization [10] and interferon [11] have been accepted in the GCT treatment, it is still necessary to perform surgical treatment thereafter. The obvious advantage of radiation therapy is the avoidance of additional surgical morbidity, while its disadvantages include local effects of radiation therapy and the potential for radiation-induced sarcoma. Leggon et al. [7] reviewed literatures of pelvic and sacral GCTs, which revealed that patients treated with radiation therapy had an incidence of 44 % of local recurrence and 12 % of radiation-induced sarcoma.

Therefore, it is quite clear that surgery plays an unparalleled role in the pelvis GCT treatments. It is widely accepted that intralesional surgery can spare nerve roots, pelvic support, and the hip and visceral structures, whereas it has a high risk of local recurrence, even for a recurrent GCT. Various adjuvant modalities have been used to supplement intralesional surgery in order for a low recurrence rate, which include the use of cytotoxic agents such as phenol [16], zinc chloride [26], ethanol [14], and physical adjuvants such as polymethylmethacrylate [13, 14, 27], cryosurgery [13], microwave inactivation [28], and a high-speed burr drill [13, 14]. Besides, a study shows that curettage based on CT classification can also lower the recurrence rate [29]. In this series, microwave inactivation had been employed for 3 patients before intralesional surgery and no recurrence occurred. It is obvious that microwave inactivation is performed before surgery other than other adjuvant treatments. The inactived tumor tissues do not have viable cells during curettage in theory. The difficulties for this method include protection of the surrounding normal tissues and complete inactivation for the whole tumor tissue. Furthermore, GCTs did not destroy the subchondral bone in the acetabular area for these 3 patients. We used polymethylmethacrylate for 2 patients in order for filling and inactivation, one of which suffered recurrence. The high-speed burr drill was employed in 2 patients to deal with residual GCT cells that may remain on the surface of the curettage cavity, and no recurrence occurred. We did not use nitrogen or other chemicals for the sake of avoiding chemical injury to the surrounding tissues from leaking adjuvants. Wide resection has been thought to minimize the chance of local recurrence, which is true for tumors in regions I and III, rather than the ones in region II. There is no doubt that wide resection is difficult to perform for large-size pelvic GCTs, especially those involving region II. An increased surgical morbidity is usual even for an experienced bone tumor surgeon.

The MSTS 93 score had been applied for patients’ functional evaluation in this study. Compared with intralesional surgery, the function of the patients who accepted wide resection appeared worse. However, it was not absolute for all patients that most patients who accepted wide resection for GCTs in regions I and III had good functions. For the GCTs involving region II or more than one region, favorable functional results were hard to achieve although some specialized prostheses had been developed to reconstruct the defect after acetabular bone resection. In this series, we preferred to use screws, titanium rods, and bone cement to reconstruct the integrity of the pelvis based on our experience. The advantages of this method include simple instruments, flexible fixed way, and relatively stable mechanical strength. The disadvantages include fixation loosening and breakup. The fixation-related complications were not observed in our patients, and most patients who accepted pelvis reconstruction could resume their routine activities within 3 months after surgery.

As we know, complications are common for pelvis tumor because of wide wound exposure, extensive soft tissue stripping, implant existence, local hematoma formation, and poor skin flap blood supply [30]. In this study, wound healing disturbance was the major complication, which occurred in 4 patients who underwent wide resection and pelvis reconstruction. Limb shortening, delayed infection, and dislocation happened in 3 patients, respectively. It is obvious that patients with GCTs in region II had a higher incidence of complication compared with those in regions I and III. For reducing complications, it is necessary to protect the skin flap and reduce soft tissue tension. Otherwise, drainage is very important for postoperative care.

It is necessary to alert readers to be aware of the limitations of this study. Firstly, the number of patients is still small although it is the largest series among the reports owing to the fact that pelvic GCTs are rare. It is hard to make any definitive statements regarding the differences of recurrences, complications, and postoperative function among different treatments without large sample statistical analysis. Secondly, this is a multi-centric retrospective study and the patients’ treatments were decided by five experienced bone tumor surgeons, respectively; consequently, the differences among surgical technologies cannot be avoided. Nevertheless, consensuses of treatments had been made by these surgeons, and postoperative situations of different treatment methods may provide valuable information for surgeons in the decision-making process. Thirdly, the minimum follow-up is short and additional local recurrences might occur with longer follow-ups. Nevertheless, 70 % of local recurrences occur within 2 years [31].

Treatment of pelvic GCTs remains a challenge for surgeons, especially for the ones involving region II. In this study, patients with GCTs that did not destroy the subchondral bone in region II achieved good functions and suffered no recurrence by undergoing microwave inactivation before intralesional surgery. Therefore, it could be an alternative treatment for some patients, although it should be determined on the basis of sufficient clinical evidence; as a consequence, a prospective study about the clinical efficacy of microwave inactivation for GCTs has already been launched by our team. It has great difficulties to achieve balance among recurrence, complications, and functions for the GCTs with benign histology and aggressive biological behavior; thus, detailed communication with patients is essential during the decision-making process.

## Conclusions

There are only a few case reports in the literature and no large numbers of clinical trials about treatment of, and research into, pelvic GCTs. In this study, low recurrence rate and favorable postoperative functional results of pelvic GCTs were obtained successfully through intralesional curettage, combined with a meticulous preoperative planning. Due to the small sample size of this study, the results should be examined cautiously. Larger, high-quality clinical trials are required to strengthen and verify these conclusions.

### Consent

The written informed consent was obtained from the patient for publication of this report and any accompanying images.
